# Identification and characterization of Ca2+-activated K+ channels in granulosa cells of the human ovary

**DOI:** 10.1186/1477-7827-7-28

**Published:** 2009-04-08

**Authors:** Matthias H Traut, Dieter Berg, Ulrike Berg, Artur Mayerhofer, Lars Kunz

**Affiliations:** 1 Institute for Cell Biology, University of Munich, Munich, Germany; 2 *Current address*: Max Planck Institute of Neurobiology, Martinsried, Germany; 3 Assisted Reproductive Technologies Bogenhausen, Munich, Germany

## Abstract

**Background:**

Granulosa cells (GCs) represent a major endocrine compartment of the ovary producing sex steroid hormones. Recently, we identified in human GCs a Ca^2+^-activated K^+ ^channel (K_Ca_) of big conductance (BK_Ca_), which is involved in steroidogenesis. This channel is activated by intraovarian signalling molecules (e.g. acetylcholine) via raised intracellular Ca^2+ ^levels. In this study, we aimed at characterizing 1. expression and functions of K_Ca _channels (including BK_Ca _beta-subunits), and 2. biophysical properties of BK_Ca _channels.

**Methods:**

GCs were obtained from in vitro-fertilization patients and cultured. Expression of mRNA was determined by standard RT-PCR and protein expression in human ovarian slices was detected by immunohistochemistry. Progesterone production was measured in cell culture supernatants using ELISAs. Single channels were recorded in the inside-out configuration of the patch-clamp technique.

**Results:**

We identified two K_Ca _types in human GCs, the intermediate- (IK) and the small-conductance K_Ca _(SK). Their functionality was concluded from attenuation of human chorionic gonadotropin-stimulated progesterone production by K_Ca _blockers (TRAM-34, apamin). Functional IK channels were also demonstrated by electrophysiological recording of single K_Ca _channels with distinctive features. Both, IK and BK_Ca _channels were found to be simultaneously active in individual GCs. In agreement with functional data, we identified mRNAs encoding IK, SK1, SK2 and SK3 in human GCs and proteins of IK and SK2 in corresponding human ovarian cells. Molecular characterization of the BK_Ca _channel revealed the presence of mRNAs encoding several BK_Ca _beta-subunits (beta2, beta3, beta4) in human GCs. The multitude of beta-subunits detected might contribute to variations in Ca^2+ ^dependence of individual BK_Ca _channels which we observed in electrophysiological recordings.

**Conclusion:**

Functional and molecular studies indicate the presence of active IK and SK channels in human GCs. Considering the already described BK_Ca_, they express all three K_Ca _types known. We suggest that the plurality and co-expression of different K_Ca _channels and BK_Ca _beta-subunits might allow differentiated responses to Ca^2+ ^signals over a wide range caused by various intraovarian signalling molecules (e.g. acetylcholine, ATP, dopamine). The knowledge of ovarian K_Ca _channel properties and functions should help to understand the link between endocrine and paracrine/autocrine control in the human ovary.

## Background

Ion channels of ovarian granulosa cells (GCs) have been identified and functionally characterized in only a few species (human, swine, chicken) ([1-3] and references in [4]). In human and porcine GCs, several channel types are involved in the physiologically important process of progesterone production [4-7]. A K_Ca _channel of large (big) conductance (BK_Ca_) in human GCs has a part in endocrine-regulated progesterone production [8]. Blocking of BK_Ca _channels results in reduction of human chorionic gonadotropin (hCG)-induced progesterone production, but does not affect basal steroidogenesis.

Moreover, BK_Ca _channels in human GCs were shown to be opened by cholinergic and oxytocinergic stimulation entailing transient membrane hyperpolarisation [8]. Acetylcholine (ACh) is produced by and also acts upon human GCs. In the human ovary, the non-neuronal cholinergic system affects several physiological functions, e.g. cell proliferation, gene transcription, and intercellular communication via gap junctions [9]. It represents just one of the local signaling systems which have been identified in recent years to complement endocrine (FSH, LH/hCG) and neuronal control of ovarian functions via autocrine/paracrine pathways. Besides ACh, other local intraovarian signaling molecules are peptide hormones (e.g. oxytocin, relaxin), catecholamines (e.g. norepinephrine, dopamine), ATP, prostaglandins, GABA and histamine. Many of these compounds (e.g. ACh, oxytocin, relaxin) exert their actions upon GCs via alteration of intracellular Ca^2+ ^levels [Ca^2+^]_i _[8-12]. In human GCs, ACh and its agonist carbachol activate muscarinic receptors (e.g. M1) and increase [Ca^2+^]_i _via Ca^2+ ^release from intracellular stores [9]. Activation of Ca^2+^-activated ion channels such as the BK_Ca _is a well-known consequence of raised [Ca^2+^]_i _[13-17].

Up to now, three K_Ca _families are known, which are classified by their single channel conductance: SK (small conductance K_Ca_), IK (intermediate conductance K_Ca_) and BK_Ca _[13,15-18]. They differ also regarding molecular and biophysical properties as well as their regulation by pharmacological compounds. For each class specific blockers exist which do not affect the two other classes. Three different apamin-sensitive SK channels were cloned (SK1, SK2, SK3), which exhibit single-channel conductances of g_sc _= 2–20 pS [18]. The IK with g_sc _= 10–80 pS is sensitive to TRAM-34 and was found in only a few non-neuronal cells, e.g. epithelial cells and erythrocytes ('Gárdos channel') [16,19-21]. The BK_Ca _channel has one of the highest known single channel conductances of g_sc _= ca. 200 pS and is sensitive to iberiotoxin (IbTx). It is the only subtype of the K_Ca _family that exhibits a pronounced voltage-dependence in addition to its Ca^2+^-sensitivity [13,14,16,22-24].

Our study aimed at the investigation of K_Ca _channels in human GCs since one member of this channel group, i.e. the BK_Ca _channel, has a part in both endocrine (i.e. role in hCG-stimulated steroidogenesis) and autocrine/paracrine pathways (i.e. cholinergic and oxytocinergic activation). The human GCs investigated originate from pre-ovulatory human follicles obtained from patients undergoing in vitro-fertilization (IVF). They represent a cell culture model of their in vivo counterparts in the antral follicle and the young active corpus luteum (CL). The study shall provide data on molecular characterization of other members of the K_Ca _channel family (SK, IK) in human GCs and their potential role in steroidogenesis. Furthermore, the Ca^2+^- and voltage-dependence of the BK_Ca _channel was determined in electrophysiological single-channel recordings. As BK_Ca _channel characteristics are affected by the type(s) of accessory β subunits present, we studied their expression in human GCs as well. There are four potential types known (β1, β2, β3, and β4), which interact with the α subunit and regulate BK_Ca _channel function regarding impact of Ca^2+ ^and voltage [25-29]. They represent also binding sites for toxins and drugs as well as phosphorylation sites [26,27,30-32].

## Methods

### Human GC preparation and culture

Human GCs were isolated from follicular aspirates of women undergoing IVF and cultured in DMEM/F12 (10% FCS; Sigma-Aldrich, Munich, Germany) under a humidified atmosphere at 37°C/5% CO_2 _[33]. Use of cells was approved by the patients and the Ethics Committee of the University of Munich. All patients were treated following standard IVF protocols and negatively diagnosed for the polycystic ovary syndrome. To account for patient-to-patient variations all studies were performed on several, randomly selected cell preparations (pooled from up to 3 patients) on different days.

### Human tissue samples

Human ovarian samples containing CL from consenting patients undergoing gynecological surgery (generously provided by C. Heiss, Klinik am Eichert, Göppingen, Germany) were fixed in Bouin's fixative and embedded in paraffin. Apart from this, we used paraffin-embedded ovarian samples with follicles from the tissue archive of the Women's Hospital in Munich, which had been taken from pre-menopausal women during autopsies [34]. The Ethics Committee of the University of Munich approved all procedures concerning use of human materials. Pathological deterioration of follicles and corpora lutea in these human ovarian samples were excluded by morphological and microscopical assessment.

### Chemicals and solutions

A stock solution of apamin (Alomone Labs, Jerusalem, Israel) was prepared in distilled water. TRAM-34 (1- [(2-Chlorophenyl)diphenylmethyl]-1H-pyrazole; Sigma-Aldrich) was dissolved in DMSO (10 mM) and final DMSO concentration in the cell culture medium did not exceed 0.01% (v/v).

### Progesterone assay

Progesterone concentrations were measured in supernatants of human GCs cultured in 24-well plates. On day three of culture, cells were treated in triplicates with the respective compounds. After 24 h the supernatants were collected and the progesterone concentrations were measured using an ELISA (DRG Instruments, Marburg, Germany). Experiments were repeated with cells from independent cell preparations (each pooled from up to 3 patients) to account for interpatient variability. After normalization to the respective control (untreated) values, data were statistically analyzed by a repeated measures ANOVA followed by Newman-Keuls multiple comparison test.

### Electrophysiology

Human GCs were grown on glass cover slips for 2–12 days and currents were recorded at room temperature by means of an EPC-9 amplifier (HEKA elektronik, Lambrecht, Germany; sample rate, 10 kHz; low pass filter, 2.5 kHz) [8]. Positive currents represent outward currents and all potentials given refer to the cytoplasmic side of the plasma membrane. Potentials were corrected for a liquid junction potential of +16 mV [35]. The extracellular solution (EC) contained (in mM) 140 NaCl, 3 KCl, 1 CaCl_2_, 1 MgCl_2_, 10 Hepes, and 10 glucose (pH 7.4). The intracellular solution (IC) contained (in mM) 130 K-gluconate, 5 NaCl, 2 EGTA, 1 MgCl_2_, and 10 Hepes (pH 7.4). Single channel currents were recorded in the inside-out configuration. To assess the Ca^2+^-sensitivity of the BK_Ca _channel the free Ca^2+ ^concentration [Ca^2+^]_i _of the IC solutions was adjusted by using varying concentrations of CaCl_2 _according to calculations performed by means of the software "Calcium" [36]. The following CaCl_2 _concentrations were used: 1.000 mM ([Ca^2+^]_i _= 100 nM), 1.870 mM ([Ca^2+^]_i _= 1 μM), 1.977 mM ([Ca^2+^]_i _= 5 μM), 1.996 mM ([Ca^2+^]_i _= 10 μM), 2.013 mM ([Ca^2+^]_i _= 20 μM), 2.100 mM ([Ca^2+^]_i _= 100 μM), and 3.000 mM ([Ca^2+^]_i _= 1 mM), respectively. To obtain channel current-voltage relationships a voltage protocol ranging from -80 mV to +90 mV in 10 mV steps was used with each potential applied for 200 ms. For evaluation of channel open probability P_o_, single channel currents were recorded at +60 mV, +70 mV, +80 mV, and +90 mV for 1600 ms in each case. For analysis, frequency histograms of current traces were calculated and the two amplitude peaks corresponding to open and closed channel state were fitted using Gaussian distributions. The single channel current amplitude was measured as differences between the two peaks. Integration of the area under the curves and division of the area under the open channel peak by the area under the entire curve yielded P_o_. In the case of two or more active individual channels in an inside-out recording, the open probability per one channel was calculated by converting the values according to the number of simultaneously active channels at each time point. [Ca^2+^]_i _values for half-maximal activation (EC_50_) were determined by fitting P_o _as a function of [Ca^2+^]_i _with a sigmoidal dose-response-curve with variable Hill slope and a fixed bottom value of zero. The voltage-dependence of P_o _was fitted using a Boltzmann sigmoidal function with the bottom value fixed to be 0 and potentials for half-maximal activation (V_50_) as well as slope values were obtained. The values are given with the respective 95% confidence interval (C.I.).

### RT-PCR

Total RNA was isolated from several human GC preparations, and reverse transcribed using Superscript-RT II (Life Technologies, Karlsruhe, Germany) in combination with either a 18-mer polydeoxythymidine primer or random hexamers of polydeoxynucleotide primers. PCR amplification was carried out with oligodeoxynucleotide primer pairs, which spanned at least one intron of the genomic sequence (except for the second BK β1 primer pair; Table 1)[37,38]. The PCR protocol consisted of 35 cycles of denaturation at 94°C (30 s), annealing at the temperatures given in Table 1 (30 s), and elongation at 72°C (45 s) using a PTC-200 Peltier Thermal Cycler (MJ Research, Bio-Rad, Munich, Germany). PCR products were separated on an agarose gel and visualized by ethidium bromide staining and ultraviolet illumination. Identity of all PCR products was verified by sequencing (Agowa, Berlin, Germany).

**Table 1:** Oligodeoxynucleotide primer pairs used for PCR amplification.

Channel subunit	Alternative nomenclature	Primer sequence 5' - 3'	GenBank accession no.	Annealing temperature	Product size	Source
BK β1	KCNMB1	Sense AAGGTCAGAGCCAAATTCCAAG	NM_004137	57°C	80 bp	ID 4758626a2
		Antisense AATAGGACGCTGGTTTCGTTC				
		Sense AGGAGGAGCTGAAGGGCAAGAAGG		57°C	309 bp	Lasergene
		Antisense AGGTGGGCCAGAAGAGGGAGAAGA				
BK β2	KCNMB2	Sense AATCACACTCCTGCGCTCATACAT	NM_181361	59°C	177 bp	Lasergene
		Antisense TCCCCGGAAGAAGTCAGGTTA				
BK β3	KCNMB3	Sense TTGCTCGGAACAACCATTCTAAA	NM_171829	57°C	155 bp	ID 25952102a2
		Antisense AGACACGGGTACTTCCCCTG				
BK β4	KCNMB4	Sense GGAAAGATGAGATTGGTTCCCAG	NM_014505	57°C	160 bp	ID 26051275a3
		Antisense AGGACCACAATGAGAACGCC				
SK1	KCNN1	Sense AGACGTGGCTCATCTACAAACA	NM_002248	59°C	149 bp	ID 25777643a3
		Antisense CTGGTCGTTCAGCTTCCCTT				
SK2	KCNN2	Sense CAAGCAAACACTTTGGTGGA	NM_021614	55°C	451 bp	[38]
		Antisense TGTTCAGGTTCCCAGGATTC				
SK3	KCNN3	Sense CATGTTTTCGTTGGCCCTGAA	NM_002249	57°C	146 bp	ID 25777650a2
		Antisense GCTCGTAGGTCATGGCTATCC				
IK	KCNN4	Sense TGTTCTACAAACATACTCGCAGG	NM_002250	64°C	134 bp	Lasergene
	SK4, K_Ca_3.1	Antisense CATGGAGTTCACTTGTTCCCG				

Primer sequences were derived from the PrimerBank (ID; ; [37], from the literature, or designed using the software Lasergene (DNAStar; see source).

### cDNA array

Total RNA of GCs cultivated for 3 days was isolated and subjected to the GEArray Q Series Human Neuroscience-1 Ion Channel and Transporter Gene Array (SuperArray Bioscience Corp., Frederick, MD). The cDNA array was analyzed by means of a chemiluminescent detection method (Roche Diagnostics, Mannheim, Germany) [39].

### Immunohistochemistry

Localization of K_Ca _α subunit proteins in human ovarian sections (5 μm) was examined according to standard procedures [34,40]. Antisera were purchased from Alomone Labs (Jerusalem, Israel) and utilized in the denoted dilutions: anti-K_Ca_3.1 (IK; rabbit anti-human; 1:200), anti-SK1 (rabbit anti-rat; 1:200), anti-SK2 (rabbit anti-rat; 1:200), anti-SK3N (rabbit anti-human; 1:500), and anti-SK3C (rabbit anti-human; 1:500). The deparaffinized sections were subjected to an additional microwave treatment for improved antigen retrieval [40] and treated with 3% H_2_O_2 _in methanol to block endogenous peroxidase. Thereafter slices were incubated at 4°C overnight with the respective antisera (containing 5% normal goat serum) and finally with goat anti-rabbit antibody (1:500). Immunoreactivity was visualized by the ABC-diaminobenzidine staining reaction (Vectastain Elite Kit, Vector Laboratories, Peterborough, UK) [34,40]. Specificity of the immunoreaction was assured by either pre-adsorption of the first antiserum with a specific peptide antigen, or its replacement by normal rabbit serum, or its complete omission.

### Cytotoxicity assays

Potential cytotoxicity of the channel blockers was evaluated by using commercial non-radioactive cell proliferation assays (CellTiter and CellTiter-Glo, both from Promega, Mannheim, Germany) [11]. Human GCs were cultured in 24- or 48-well plates and treated in triplicates for 24 h on day 3 of culture with the substances applied for progesterone measurements.

### Data analysis

Data were analyzed and depicted using Prism 4 (GraphPad Software, San Diego, California) and SigmaPlot 10 (SPSS, Chicago, Illinois). Data represent means ± SEM if not stated otherwise.

## Results

### Role of K_Ca _channels in steroidogenesis in human GCs

**Figure 1:**
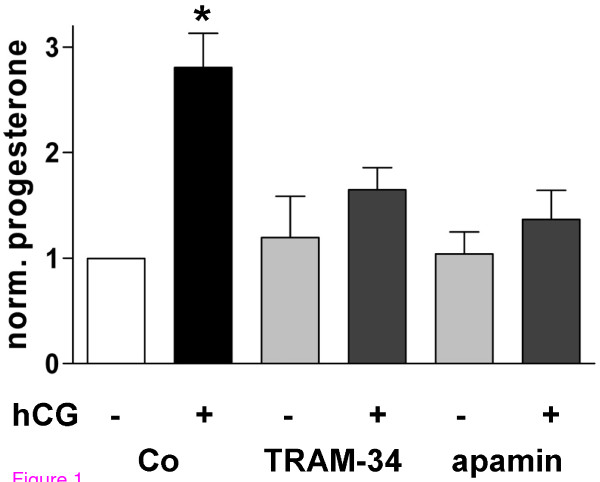
**Opening of K_Ca _channels is required for hCG-stimulated steroidogenesis in human GCs**. Blocking of IK (1 μM TRAM-34) and SK channels (100 nM apamin) significantly attenuated hCG (10 IU/ml)-induced progesterone production (P_ANOVA _= 0.0038). According to Newman-Keuls multiple comparison test only the hCG-treated group (*) is significantly different (P < 0.01 compared to all groups), whereas there is no difference between all other groups (P > 0.05). Data represent means ± SEM (n = 3 experiments using independent cell preparations treated on the 3^rd ^day of culture for 24 h).

Functionality of BK_Ca _channels is known to be necessary for steroidogenesis in human GCs [8]. Blocking of IK channels by TRAM-34 (1 μM) and of SK channels by apamin (100 nM) had an inhibitory action on hCG (10 IU/ml)-induced progesterone production as well (Figure 1). The blockers had no effect on basal progesterone production. This casts cytotoxic actions of the blockers into doubt. In addition, neither two different proliferation/cytotoxicity assays nor inspection of the cells by light and electron microscopy showed any detrimental alterations caused by the K_Ca _blockers (data not shown).

### Expression of different K_Ca _channel families in human GCs

**Figure 2:**
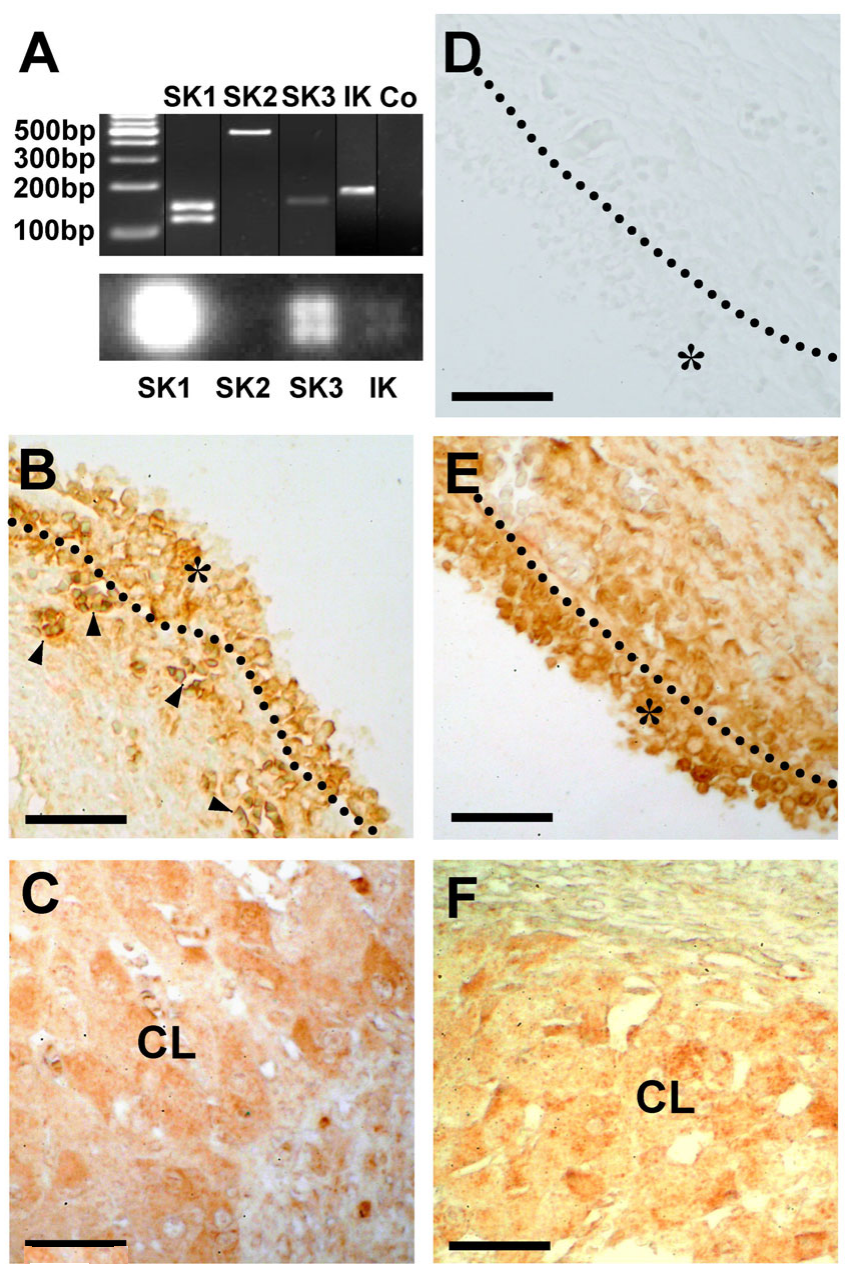
**Various K_Ca _channels are expressed in human GCs**. (*A*) Cultured GCs express mRNAs encoding SK and IK channels as shown by RT-PCR (top) and cDNA array techniques (bottom). In negative RT-PCR controls (Co) template was replaced by water. (*B*, *C*) Expression of SK2 α subunit protein in follicular (*B*) and luteal GCs (*C*) of the human ovary. (*D*) Example of a control experiment (omission of first antiserum) using a slice of the same ovarian sample as in *B *and *E*. (*E*, *F*) Expression of IK α subunit protein in follicular (*E*) and luteal GCs (*F*) of the human ovary. The layer of follicular GCs (*) is delimited by a dotted line (*B*, *D*, *E*). Arrowheads in *B *label small blood vessels with erythrocytes, which are immunopositive as well. CL, Corpus luteum. Bars, 50 μm.

The presence of mRNAs coding for all known types of SK channels and the IK channel was demonstrated by cDNA arrays (except for SK2) and by RT-PCR followed by sequencing (Figure 2A). In case of SK1 two PCR products were found and sequenced, which both correspond to the respective channel. They differ with regard to presence or absence of exon 9 (the primers match sequences in exon 8 and 10, respectively) and are, thus, most likely yet unknown splicing variants. IK and SK2 α subunit proteins are expressed in GCs of the human ovary (Figure 2). Expression of SK1 and SK3 α subunits cannot be unambiguously appraised since the antisera used for immunohistochemistry produced unspecific (e.g. nuclear) staining (data not shown).

**Figure 3:**
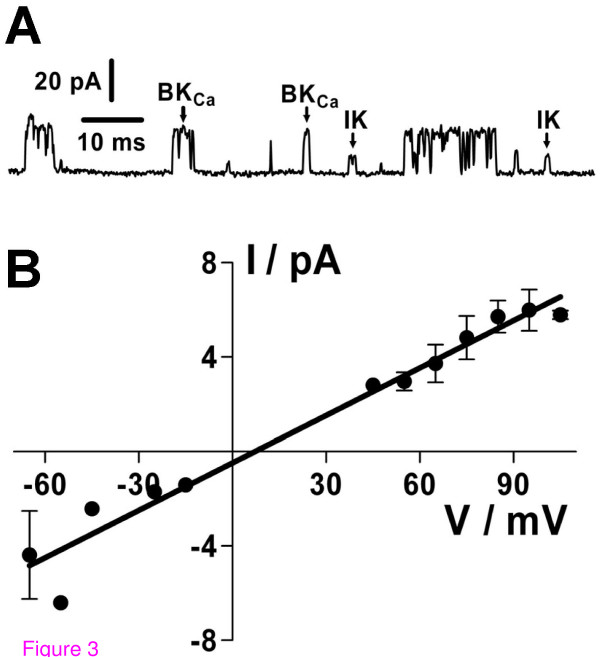
**IK channels are functional in inside-out recordings of human GCs**. (*A*) Current trace exhibiting simultaneous activity of IK and BK_Ca _in the same membrane patch (recorded at a holding potential of +90 mV). (*B*) The IK current-voltage relationship yielded a single-channel conductance of 67 ± 5 pS and a reversal potential of 7 mV (n = 5 recordings on individual cells). Data points represent means ± SEM (error bars only depicted when more than two values were available). All currents were recorded under symmetrical high K^+^-gluconate concentrations.

In electrophysiological recordings in the inside-out configuration, a second type of K_Ca _channel besides the BK_Ca _was recorded at [Ca^2+^]_i _> 1 μM (Figure 3). This channel is most likely the IK channel because of its characteristic intermediate single-channel conductance of 67 ± 5 pS and a reversal potential of V_0 _= 7 mV under symmetrical K^+^-gluconate concentrations (n = 5; Figure 3B), its increasing P_o _with rising [Ca^2+^]_i _(data not shown), and because K^+ ^was the only permeant ion present in sufficiently high concentrations on both sides of the membrane. The maxiCl can be excluded due to the low symmetrical Cl^-^-concentrations of 9 mM. In almost all recordings exhibiting the IK channel, at least one BK_Ca _channel was simultaneously active (Figure 3A).

### Ca^2+^- and voltage-dependence of BK_Ca _channels in human GCs

**Figure 4:**
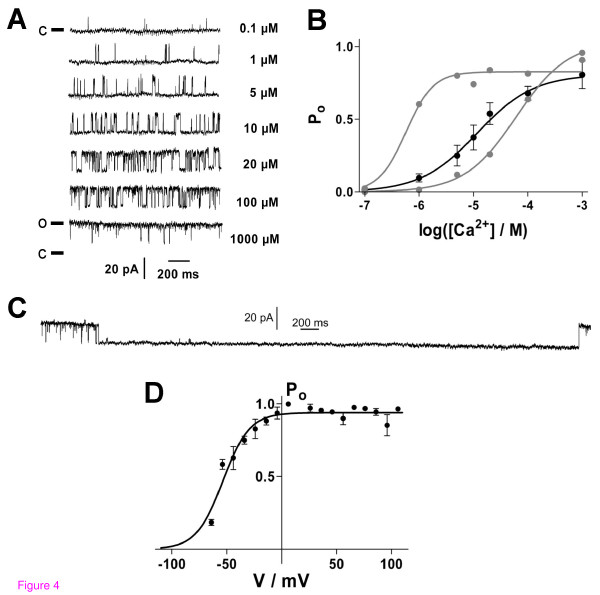
**Electrophysiological characterization of Ca^2+^- and voltage-dependence of BK_Ca _channels in human GCs (inside-out recordings)**. (*A*) BK_Ca _current traces recorded at +60 mV and varying [Ca^2+^]_i _(right). The closed channel state (c) corresponds to the respective lower current level (o, open state). (*B*) Ca^2+^-dependence of the channel open probability, P_o_, at +70 mV. Data (black circles) represent means ± SEM of recordings from 30 cells and were fitted by a sigmoidal dose-response function with the bottom value fixed to be 0. EC_50 _= 12 μM, Hill-slope = +0.8 μM (n = 30). Gray circles and fit curves show data from two individual recordings that represent extrema of Ca^2+^-dependence of P_o_. (*C*) BK_Ca _channel inactivation at [Ca^2+^]_i _= 1 mM and +70 mV. (*D*) Voltage-dependence of P_o _at [Ca^2+^]_i _= 1 mM (V_50 _= -54 mV, n = 4). Data were fitted by a Boltzmann sigmoidal function with bottom value fixed to be 0.

BK_Ca _channels recorded in the inside-out configuration exhibited a characteristic single channel conductance in the range of 200 pS. Channel open probability P_o _increased with both elevating [Ca^2+^]_i _and voltage (Figure 4). The Ca^2+^-dependence of the BK_Ca _was evaluated by fitting P_o _as a function of [Ca^2+^]_i _with a sigmoidal dose-response curve (Figure 4B). The [Ca^2+^]_i _for half-maximal activation were EC_50 _(60 mV) = 20 μM (9 to 41 μM; n = 30), Hill slope = 1.0 μM (0.5 to 1.5 μM), and EC_50 _(70 mV) = 12 μM (6 to 24 μM, n = 30), slope = 0.8 μM (0.3 to 1.4 μM). Comparing individual cells, heterogeneity of EC_50 _values – even in the same cell preparation – was observed. The values covered a range of 2 to 118 μM (at +60 mV; n = 8) and of 1 to 91 μM (at +70 mV; n = 9), respectively. At high [Ca^2+^]_i _(1 mM) and high positive potentials (+70 mV) inactivation of BK_Ca _channels was observed for time periods ranging from several hundred ms to several s (Figure 4C). Periods of inactivation longer than 500 ms were not considered when calculating P_o_; only the trace before onset of inactivation was evaluated. The voltage-dependence of the BK_Ca _channel was determined at high [Ca^2+^]_i _because at lower [Ca^2+^]_i _the channel was not active at negative potentials. By fitting the data with a Boltzmann sigmoidal function a potential for half-maximal activation of V_50 _= -54 mV (-56 to -51 mV, 95%-C.I.) and a slope factor of +12 mV (+9 to +15 mV) were observed at [Ca^2+^]_i _= 1 mM (n = 4; Figure 4D).

### Expression of BK_Ca _β subunits in human GCs

**Figure 5:**
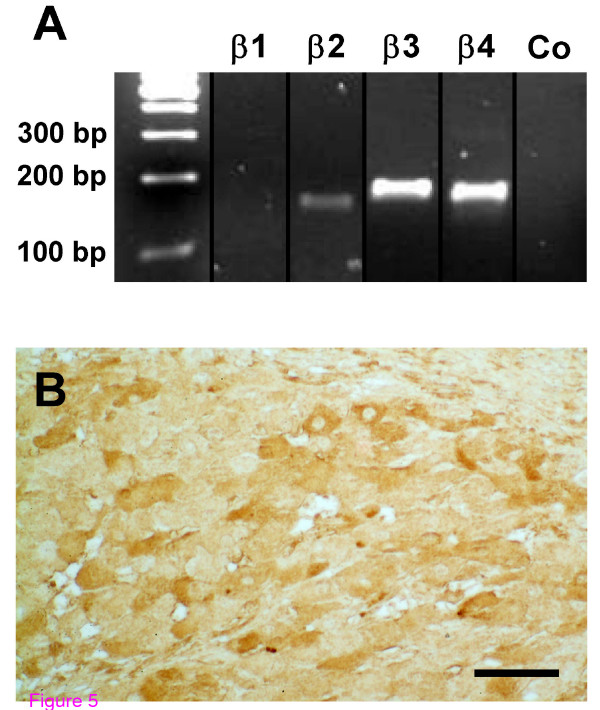
**Expression of BK_Ca _β subunits**. (*A*) In human GCs, mRNAs coding for several β subunits were detected. In negative controls (Co) template was replaced by water. (*B*) BK_Ca _β4 subunit protein is present in GCs of human CL. Bar, 50 μm.

By RT-PCR and subsequent sequencing, we identified mRNAs encoding the BK_Ca _subunits β2, β3, and β4 in human GCs (Figure 5A). The β1 subunit was not detected using two different primer pairs, which amplified the β1 subunit in positive control tissues (ovary, prostate, testis, colon, heart, and lung; data not shown). The BK_Ca _β4 subunit protein was also found in endocrine cells of the human CL by means of immunohistochemistry (Figure 5B).

## Discussion

In the present communication we report that human GCs possess in addition to the BK_Ca _other functional K_Ca _channels. The detection of mRNAs encoding the intermediate-conductance K_Ca _(IK) as well as all three known types of small-conductance K_Ca _(SK1, SK2, SK3) points at a complex K_Ca _repertoire. The finding of mRNA alone can be misleading as was shown in a study on glioma cells in which mRNAs for all K_Ca _channels were detected, although only BK_Ca _channels were functional [41]. However, in human GCs all three classes of K_Ca _channels are functional, because specific blockers attenuated hCG-stimulated steroidogenesis. The IK blocker TRAM-34 was recently reported to inhibit nonselective cation channels as well [42], but is still one of the most accepted pharmacological tools to block IK channels. We cannot definitely exclude a role of nonselective cation channels. However, as they were not found in human GCs so far and because we presented further molecular proof of IK presence, we interpret the TRAM-34 action as blockage of IK channels. In addition, single K_Ca _channels were recorded which we identified as IK based on Ca^2+^-dependence and single channel conductance. A role of IK and SK channels in vivo is assumed because the corresponding proteins (IK, SK2) were detected – like BK_Ca _[8] – in endocrine cells of the human ovary. Despite mRNAs coding for all three known SK channels were found, we can only conclude that at least one of them is functional due to the impact of the SK blocker apamin on hCG-induced steroidogenesis. As the SK2 protein was detected in human ovarian slices it is likely that this type (and probably others) is present in cultured human GCs.

Besides their role in endocrine stimulated steroidogenesis, K_Ca _channels in human GCs represent a link to local regulatory systems of the ovary operating via signaling molecules (ACh, oxytocin, relaxin, norepinephrine, dopamine, ATP) that are known to alter [Ca^2+^]_i _[10-12]. Therefore, and because the BK_Ca _is the best studied K_Ca _channel, we assessed its Ca^2+^- and voltage-dependence by means of single-channel recordings. The BK_Ca _channel was half-activated at about 20 μM [Ca^2+^]_i _at +60 mV. An increase of [Ca^2+^]_i _by one order of magnitude is known to shift the potential V_50 _necessary for half-maximal activation in several other cell types by about 50 mV (30 to 94 mV) to more negative values [43-45]. At a global [Ca^2+^]_i _= 1 μM that can be reached by stimulation with ACh, oxytocin, or relaxin [8-10], the potential V_50 _necessary for half-maximal activation can be estimated to equal +100 mV. Although this represents a rather non-physiological voltage value, BK_Ca _channels can be activated under physiological conditions in human GCs since its specific blocking with IbTx attenuates hCG-stimulated progesterone production [8]. This apparent discrepancy might be explained by the fact that open probabilities much lower than 50% could account for physiologically sufficient macroscopic (whole-cell) currents. In addition, during stimulation local rises in [Ca^2+^]_i _in close proximity to the channel might be much higher than the monitored overall cytoplasmic elevation of [Ca^2+^]_i_, and thereby BK_Ca _channels can be opened at more physiological voltages. Pronounced differences in alteration of global [Ca^2+^]_i _monitored by standard techniques and of local [Ca^2+^]_i _were described in other cell types [46,47].

The variability of EC_50 _values for Ca^2+^-sensitivity of the BK_Ca _channel over two orders of magnitude could be due to different β subunits present since they are known to be important modulators of Ca^2+^- and voltage-sensitivity of the channel [25-32,48]. Similar variations of EC50 and half-maximal activation voltages were reported for BK_Ca _in other cell types [13,15,43,45,49-51]. Therefore, we have studied the BK_Ca _β subunit expression in human GCs and found mRNAs encoding the accessory subunits β2, β3, and β4, but not β1. Reports on the presence of β2, β3 and β4 mRNAs in material from whole human ovaries can now be better interpreted in terms of a GCs contribution to the mRNA findings [29,31].

The consequences of BK_Ca _β subunit expression pattern for channel function in human GCs are difficult to compare with studies in cellular model systems expressing only one type of β subunit. Nevertheless, the β subunit repertoire found on the mRNA level might help to explain our observations. The recorded inactivation of BK_Ca _channels at high positive potentials and at high free [Ca^2+^]_i _was reported to occur in the presence of β2 and/or β3 subunits [26-28,52,53]. The absence of the β1 subunit might explain why oestrogens do not activate the BK_Ca _in human GCs [8]; in contrast to myometrial smooth muscle in which activation by oestrogens was ascribed to the presence of β1 [54,55]. The presence of β4 should introduce IbTx-resistance to BK_Ca _channels [32]. However, the fact that IbTx blocks both BK_Ca _whole-cell currents and hCG-stimulated progesterone production points at a more complex picture, i.e. at least a proportion of the BK_Ca _channels in human GCs is IbTx-sensitive and, thus, probably contains not only β4 [8].

The simultaneous presence of different K_Ca _types is known from other cell types [18]. But what could be the cellular relevance of the ostensible redundancy to have different K_Ca _channels? They differ regarding regulation, biophysical properties as well as Ca^2+ ^sensitivity with IK and SK channels being activated at lower [Ca^2+^]_i _than the BK_Ca _[17,18]. In addition, the BK_Ca _is voltage-sensitive in contrast to other K_Ca _channels [13,14,16,24], which would allow differentiated responses to the same Ca^2+ ^signals at varying membrane potentials. Concerning the multitude of K_Ca _channels in human GCs the question arises whether each individual GC expresses all identified K_Ca _channel subunits in parallel. The RT-PCR and progesterone production experiments can provide no answers about single cells. But single channel recordings revealed that at least BK_Ca _and IK channels can be present in the plasma membrane of the same individual cells. However, it is very likely that individual GCs can exhibit a varying K_Ca _repertoire and that GC subpopulations might exist regarding the expression of β subunits. Immunohistochemical results are in favor of such an assumption, since for IK, SK2, and β4, the degree of immunostaining in GCs of the human CL varies. The variations in Ca^2+^-sensitivity observed in single BK_Ca _channel recordings might also reflect BK_Ca _heterogeneity in single GCs and/or between individual GCs.

## Conclusion

In summary, we found expression (in vitro, ex vivo) of several classes of K_Ca _channels in human GCs, which are all involved in gonadotropin-stimulated sex steroid hormone production. The presence of different K_Ca _channels and the observed heterogeneity in Ca^2+^-sensitivity of the BK_Ca _channel, which is probably due to expression of various β subunits, could allow finely tuned and differentiated cellular responses over a wide [Ca^2+^]_i _range. The question of existence of GCs subpopulations regarding K_Ca _channels and BK_Ca _β subunits has to be studied in the future to understand cellular processes on the level of individual GCs. The rich instrumentation of Ca^2+^-dependent channels might be seen in relation to the abundance of intraovarian signaling molecules (e.g. ACh, ATP, dopamine, oxytocin, relaxin) acting via raised Ca^2+ ^levels. Therefore, we suggest that this channel group has a part in mediating the conjunction between endocrine (hCG, LH) and local ovarian signaling systems.

## Abbreviations

ACh: acetylcholine; BK_Ca_: big conductance K_Ca_; [Ca^2+^]_i_: intracellular Ca^2+ ^concentration; C.I.: confidence interval; CL: Corpus luteum; EC_50_: [Ca^2+^]_i _for half-maximal activation; GC: granulosa cell; g_sc_: single-channel conductance; hCG: human chorionic gonadotropin; IbTx: iberiotoxin; IK: intermediate conductance K_Ca_; IVF: in vitro-fertilization; K_Ca_: Ca^2+^-activated K^+ ^channel; P_o_: channel open probability; SK: small conductance K_Ca_; TRAM-34: 1- [(2-Chlorophenyl)diphenylmethyl]-1H-pyrazole; V_50_: potential for half-maximal activation.

## Competing interests

The authors declare that they have no competing interests.

## Authors' contributions

MHT performed most of the experiments, was involved in conception of the study, contributed to analysis and interpretation of the data and to writing of the manuscript. DB and UB provided human GCs and were involved in study design. AM conceived of the study and contributed to interpretation of the data and to writing of the manuscript. LK conceived of the study, coordinated the experiments, contributed to analysis and interpretation of the data and to writing of the manuscript. All authors read and approved the final manuscript.

## References

[B1] Kusaka M, Tohse N, Nakaya H, Tanaka T, Kanno M, Fujimoto S (1993). Membrane currents of porcine granulosa cells in primary culture: characterization and effects of luteinizing hormone. Biol Reprod.

[B2] Manikkam M, Li Y, Mitchell BM, Mason DE, Freeman LC (2002). Potassium channel antagonists influence porcine granulosa cell proliferation, differentiation, and apoptosis. Biol Reprod.

[B3] Mason DE, Mitchell KE, Li Y, Finley MR, Freeman LC (2002). Molecular basis of voltage-dependent potassium currents in porcine granulosa cells. Mol Pharmacol.

[B4] Mayerhofer A, Kunz L (2006). Ion channels of primate ovarian endocrine cells: identification and functional significance. Expert Rev Endocrinol Metab.

[B5] Olivero P, Leiva-Salcedo E, Devoto L, Stutzin A (2008). Activation of Cl- channels by human chorionic gonadotropin in luteinized granulosa cells of the human ovary modulates progesterone biosynthesis. Endocrinology.

[B6] Platano D, Magli MC, Ferraretti AP, Gianaroli L, Aicardi G (2005). L- and T-type voltage-gated Ca2+ channels in human granulosa cells: functional characterization and cholinergic regulation. J Clin Endocrinol Metab.

[B7] Li Y, Ganta S, von Stein FB, Mason DE, Mitchell BM, Freeman LC (2003). 4-aminopyridine decreases progesterone production by porcine granulosa cells. Reprod Biol Endocrinol.

[B8] Kunz L, Thalhammer A, Berg FD, Berg U, Duffy DM, Stouffer RL, Dissen GA, Ojeda SR, Mayerhofer A (2002). Ca2+-activated, large conductance K+ channel in the ovary: identification, characterization, and functional involvement in steroidogenesis. J Clin Endocrinol Metab.

[B9] Mayerhofer A, Kunz L (2005). A non-neuronal cholinergic system of the ovarian follicle. Ann Anat.

[B10] Mayerhofer A, Engling R, Stecher B, Ecker A, Sterzik K, Gratzl M (1995). Relaxin triggers calcium transients in human granulosa-lutein cells. Eur J Endocrinol.

[B11] Rey-Ares V, Lazarov N, Berg D, Berg U, Kunz L, Mayerhofer A (2007). Dopamine receptor repertoire of human granulosa cells. Reprod Biol Endocrinol.

[B12] Tai CJ, Kang SK, Leung PC (2001). Adenosine triphosphate-evoked cytosolic calcium oscillations in human granulosa-luteal cells: role of protein kinase C. J Clin Endocrinol Metab.

[B13] Latorre R, Oberhauser A, Labarca P, Alvarez O (1989). Varieties of calcium-activated potassium channels. Annu Rev Physiol.

[B14] Latorre R, Brauchi S (2006). Large conductance Ca2+-activated K+ (BK) channel: activation by Ca2+ and voltage. Biol Res.

[B15] McManus OB (1991). Calcium-activated potassium channels: regulation by calcium. J Bioenerg Biomembr.

[B16] Vergara C, Latorre R, Marrion NV, Adelman JP (1998). Calcium-activated potassium channels. Curr Opin Neurobiol.

[B17] Kaczorowski GJ, Garcia ML (1999). Pharmacology of voltage-gated and calcium-activated potassium channels. Curr Opin Chem Biol.

[B18] Pedarzani P, Stocker M (2008). Molecular and cellular basis of small- and intermediate-conductance, calcium-activated potassium channel function in the brain. Cell Mol Life Sci.

[B19] Maher AD, Kuchel PW (2003). The Gardos channel: a review of the Ca2+-activated K+ channel in human erythrocytes. Int J Biochem Cell Biol.

[B20] Bissonnette JM (2002). The role of calcium-activated potassium channels in respiratory control. Respir Physiol Neurobiol.

[B21] Wei AD, Gutman GA, Aldrich R, Chandy KG, Grissmer S, Wulff H (2005). International Union of Pharmacology. LII. Nomenclature and molecular relationships of calcium-activated potassium channels. Pharmacol Rev.

[B22] Salkoff L, Butler A, Ferreira G, Santi C, Wei A (2006). High-conductance potassium channels of the SLO family. Nat Rev Neurosci.

[B23] Ghatta S, Nimmagadda D, Xu X, O'Rourke ST (2006). Large-conductance, calcium-activated potassium channels: structural and functional implications. Pharmacol Ther.

[B24] Gribkoff VK, Starrett JE, Dworetzky SI (2001). Maxi-K potassium channels: form, function, and modulation of a class of endogenous regulators of intracellular calcium. Neuroscientist.

[B25] Nimigean CM, Magleby KL (1999). The beta subunit increases the Ca2+ sensitivity of large conductance Ca2+-activated potassium channels by retaining the gating in the bursting states. J Gen Physiol.

[B26] Torres YP, Morera FJ, Carvacho I, Latorre R (2007). A marriage of convenience: beta-subunits and voltage-dependent K+ channels. J Biol Chem.

[B27] Orio P, Rojas P, Ferreira G, Latorre R (2002). New disguises for an old channel: MaxiK channel beta-subunits. News Physiol Sci.

[B28] Uebele VN, Lagrutta A, Wade T, Figueroa DJ, Liu Y, McKenna E, Austin CP, Bennett PB, Swanson R (2000). Cloning and functional expression of two families of beta-subunits of the large conductance calcium-activated K+ channel. J Biol Chem.

[B29] Jiang Z, Wallner M, Meera P, Toro L (1999). Human and rodent MaxiK channel beta-subunit genes: cloning and characterization. Genomics.

[B30] Dworetzky SI, Boissard CG, Lum-Ragan JT, McKay MC, Post-Munson DJ, Trojnacki JT, Chang CP, Gribkoff VK (1996). Phenotypic alteration of a human BK (hSlo) channel by hSlobeta subunit coexpression: changes in blocker sensitivity, activation/relaxation and inactivation kinetics, and protein kinase A modulation. J Neurosci.

[B31] Behrens R, Nolting A, Reimann F, Schwarz M, Waldschutz R, Pongs O (2000). hKCNMB3 and hKCNMB4, cloning and characterization of two members of the large-conductance calcium-activated potassium channel beta subunit family. FEBS Lett.

[B32] Meera P, Wallner M, Toro L (2000). A neuronal beta subunit (KCNMB4) makes the large conductance, voltage- and Ca2+-activated K+ channel resistant to charybdotoxin and iberiotoxin. Proc Natl Acad Sci USA.

[B33] Agoston A, Kunz L, Krieger A, Mayerhofer A (2004). Two types of calcium channels in human ovarian endocrine cells: involvement in steroidogenesis. J Clin Endocrinol Metab.

[B34] Mayerhofer A, Hemmings HC, Snyder GL, Greengard P, Boddien S, Berg U, Brucker C (1999). Functional dopamine-1 receptors and DARPP-32 are expressed in human ovary and granulosa luteal cells in vitro. J Clin Endocrinol Metab.

[B35] Barry PH, Lynch JW (1991). Liquid junction potentials and small cell effects in patch-clamp analysis. J Membr Biol.

[B36] Fohr KJ, Warchol W, Gratzl M (1993). Calculation and control of free divalent cations in solutions used for membrane fusion studies. Methods Enzymol.

[B37] Wang X, Seed B (2003). A PCR primer bank for quantitative gene expression analysis. Nucleic Acids Res.

[B38] Platoshyn O, Remillard CV, Fantozzi I, Mandegar M, Sison TT, Zhang S, Burg E, Yuan JX (2004). Diversity of voltage-dependent K+ channels in human pulmonary artery smooth muscle cells. Am J Physiol Lung Cell Mol Physiol.

[B39] Fritz S, Kunz L, Dimitrijevic N, Grunert R, Heiss C, Mayerhofer A (2002). Muscarinic receptors in human luteinized granulosa cells: activation blocks gap junctions and induces the transcription factor early growth response factor-1. J Clin Endocrinol Metab.

[B40] Fritz S, Wessler I, Breitling R, Rossmanith W, Ojeda SR, Dissen GA, Amsterdam A, Mayerhofer A (2001). Expression of muscarinic receptor types in the primate ovary and evidence for nonneuronal acetylcholine synthesis. J Clin Endocrinol Metab.

[B41] Weaver AK, Bomben VC, Sontheimer H (2006). Expression and function of calcium-activated potassium channels in human glioma cells. Glia.

[B42] Schilling T, Eder C (2007). TRAM-34 inhibits nonselective cation channels. Pflugers Arch.

[B43] Womack MD, Khodakhah K (2002). Characterization of large conductance Ca2+-activated K+ channels in cerebellar Purkinje neurons. Eur J Neurosci.

[B44] Rothberg BS, Magleby KL (2000). Voltage and Ca2+ activation of single large-conductance Ca2+-activated K+ channels described by a two-tiered allosteric gating mechanism. J Gen Physiol.

[B45] Lara J, Acevedo JJ, Onetti CG (1999). Large-conductance Ca2+-activated potassium channels in secretory neurons. J Neurophysiol.

[B46] Lohmann C, Wong RO (2005). Regulation of dendritic growth and plasticity by local and global calcium dynamics. Cell Calcium.

[B47] Schneggenburger R, Neher E (2005). Presynaptic calcium and control of vesicle fusion. Curr Opin Neurobiol.

[B48] Brenner R, Perez GJ, Bonev AD, Eckman DM, Kosek JC, Wiler SW, Patterson AJ, Nelson MT, Aldrich RW (2000). Vasoregulation by the beta1 subunit of the calcium-activated potassium channel. Nature.

[B49] Lovell PV, James DG, McCobb DP (2000). Bovine versus rat adrenal chromaffin cells: big differences in BK potassium channel properties. J Neurophysiol.

[B50] Sugihara I (1994). Calcium-activated potassium channels in goldfish hair cells. J Physiol.

[B51] Tanaka Y, Meera P, Song M, Knaus HG, Toro L (1997). Molecular constituents of maxi KCa channels in human coronary smooth muscle: predominant alpha + beta subunit complexes. J Physiol.

[B52] Hicks GA, Marrion NV (1998). Ca2+-dependent inactivation of large conductance Ca2+-activated K+ (BK) channels in rat hippocampal neurones produced by pore block from an associated particle. J Physiol.

[B53] Xia XM, Ding JP, Lingle CJ (2003). Inactivation of BK channels by the NH2 terminus of the beta2 auxiliary subunit: an essential role of a terminal peptide segment of three hydrophobic residues. J Gen Physiol.

[B54] Benkusky NA, Korovkina VP, Brainard AM, England SK (2002). Myometrial maxi-K channel beta1 subunit modulation during pregnancy and after 17beta-estradiol stimulation. FEBS Lett.

[B55] Dick GM, Sanders KM (2001). (Xeno)estrogen sensitivity of smooth muscle BK channels conferred by the regulatory beta1 subunit: a study of beta1 knockout mice. J Biol Chem.

